# A brief version of the Attitudes to Ageing Questionnaire for older Chinese adults: development and psychometric evaluation

**DOI:** 10.1186/s40359-024-01691-z

**Published:** 2024-04-01

**Authors:** Lin Gao, Ken Laidlaw, Dahua Wang

**Affiliations:** 1 Institute of Developmental Psychology, Beijing Normal University, No.19 Xinjiekouwai Street, 100875 Beijing, China; 2 Department of Psychology, University of Exeter, Exeter, UK

**Keywords:** Attitudes toward aging, Scale development, Ageism, Older people

## Abstract

**Background:**

Positive attitudes toward aging are considered essential for achieving psychological well-being in later life. However, there is currently a lack of a concise and comprehensive measurement tool specifically designed to assess attitudes toward aging among the elderly population in China. To address this gap, the present study aimed to develop a brief version of the Attitudes to Ageing Questionnaire tailored to older Chinese individuals and evaluate its psychometric properties.

**Methods:**

Initially, a sample of community-dwelling older adults (Sample 1: *n* = 442, aged 60–88) was utilized to establish a new scale format. Subsequently, two convenience samples (Sample 2: *n* = 311, aged 60–90; Sample 3: *n* = 164, aged 60–89) were employed to evaluate the psychometric properties of this scale, including factor structure, internal consistency, test-retest reliability, convergent validity, and discriminant validity.

**Results:**

We selected 12 items from the original questionnaire to create the brief scale. The brief scale maintained the three-factor structure of the full-format version, encompassing psychosocial loss, physical change, and psychological growth, and demonstrated adequate psychometric properties.

**Conclusions:**

This development process shortens the administration time of the questionnaire while avoiding excessive loss of information. The newly developed scale serves as a reliable and valid assessment tool for measuring attitudes toward aging among older Chinese individuals and is well-suited for implementation in large-scale surveys that utilize an extensive array of questionnaires. This tool can be applied to assessing the effectiveness of interventions aimed at eliminating ageism.

**Supplementary Information:**

The online version contains supplementary material available at 10.1186/s40359-024-01691-z.

## Background

Older adults often find themselves in a disadvantaged position. For example, ageism, characterized by negative stereotypes, prejudice, and discrimination against older adults [1], can have adverse effects on the physical and mental health of older individuals [2–4]. Social stigmatization due to being perceived as a financial burden on social well-fare can lead to blame and isolation. Moreover, in an era of technological innovation and cultural change, older adults are more likely to experience maladaptation rather than benefit. During the COVID-19 pandemic, the situation for older adults has further worsened [5–8]. These factors may collectively contribute to negative perceptions of aging among older adults, thereby impairing their psychological well-being.

China is currently facing a “senior tsunami” as the proportion of individuals aged 60 and above has risen from 13.26 to 18.70% between 2010 and 2020, with further growth expected in the next decade [9]. The rapid increase in the elderly population may lead to a rise in the negative age-related experiences. In response, the Chinese government plans to improve the perceptions of later life among the expanding elderly population through a series of policy interventions [10]. To assess the effectiveness of policies, it is essential to develop a brief and comprehensive assessment tool that evaluates how older Chinese adults perceive the aging process.

### Attitudes toward aging

Attitudes toward aging refer to the general perception of aging as well as older adults’ understanding and expectations of their own aging process and later life [11]. A relevant and popular field of study is research on age stereotypes. As people age, their internalized negative depictions of old age become self-relevant, resulting in a pessimistic self-perception of aging [4]. Once they are aware that their behaviors may confirm the negative expectations of the group to which they belong, a stereotype threat occurs [12]. These negative perceptions of aging have consistently been demonstrated to have detrimental effects on the cognitive performance and health outcomes of older adults [3, 13, 14]. Conversely, extensive research has established the essential role of nurturing optimistic attitudes toward aging for enhancing well-being in late adulthood. This encompasses experiencing a higher quality of life [15, 16], fewer depressive symptoms [17, 18], lower levels of anxiety [19], and better physical health [20, 21]. Moreover, positive attitudes toward aging are linked to other psychological resources, such as self-efficacy beliefs [22].

Hence, conducting comprehensive and systematic investigations into the attitudes of older adults toward aging carries multiple implications. First, they contribute to a better understanding of older individuals’ perceptions of their own age, thereby shedding light on their psychological status, emotions, attitudes, and expectations toward life. Second, by assessing attitudes toward aging, we can predict older adults’ levels of well-being and mental health. Last but not least, gaining insights into the attitudes and beliefs of older adults enables policymakers and intervention designers to effectively address the needs of this population, foster active aging, and improve their quality of life.

### Existing assessment tools

In previous research, various assessment tools, including the Kogan’s Attitudes Toward Old People Scale [23], the Fraboni Scale of Ageism [24], and the Anxiety about Aging Scale [25], have been utilized to measure attitudes toward older individuals or expectations about aging. Nevertheless, these measurement tools were not explicitly designed for older adults and had limitations in assessing perceptions of aging from their own perspective. In addition, Lawton [26] developed and revised the Attitude Toward Own Aging tool, which serves as a commonly used measurement in existing literature. This tool, however, is a subscale of the Philadelphia Geriatric Center Morale Scale, with limited contribution to the multidimensional concept of attitudes toward the aging process. Moreover, Barker et al. [27] developed the Aging Perceptions Questionnaire to evaluate attitudes toward aging across seven domains, and the Chinese version of this questionnaire was subsequently validated with adequate psychometric properties [28]. Nonetheless, the statements used are primarily general and do not specify particular functional maintenance and changes within the aging process. According to the life-span development theory [29], the aging process involves both growth and decline concurrently occurring in various domains, such as physical function, social connectedness, and psychological transitions. These factors interact with each other but also contribute to relatively independent experiences of aging, as observed in the paradox of aging [30]. Consequently, evaluating attitudes toward aging demands a multidimensional and multidirectional framework. This tool ideally provides insights into how older individuals perceive specific life contexts, thereby informing policy-making objectives.

To tackle this issue, Laidlaw et al. developed the Attitudes to Ageing Questionnaire (AAQ) [31], a comprehensive instrument that assesses attitudes toward aging using a multidimensional framework. The research team formulated two types of item expression, capturing both general aging attitudes and self-relevant attitudes. These items encompass perceptions of age-related gain and loss and are categorized into three subscales: psychosocial loss (PL), physical change (PC), and psychological growth (PG). The AAQ has been adapted into multiple versions across various cultural backgrounds, including Brazilian [32], Chinese [33], Norwegian [34], Spanish [35], French [36], Farsi [37], Malay [38], and Portuguese [39], and has shown sufficient cross-cultural validity. However, the full version of the AAQ is rarely utilized in surveys that use a large battery of questionnaires due to its length (24 items), which can be time-consuming to complete.

In this regard, Laidlaw et al. developed a shortened format of the AAQ (AAQ-SF) [40], which reduced the questionnaire length to 12 items. However, the item selection process relied on a sample predominantly representing Western cultural backgrounds (66.10% from European countries, 8.93% from North America, and 6.76% from Australia), with a small percentage from East-Asia (3.38% from Japan). Consequently, some cultural elements specific to East Asian societies, such as the reverence for wisdom of older individuals (e.g., item 4 “Wisdom comes with age”), were omitted. In Chinese culture, older people are often considered the embodiment of wisdom, and the prevalence of filial piety has bestowed upon older adults a revered social status [41–43]. However, recent cross-cultural studies have not confirmed that East-Asian countries show greater respect for the older people than Western countries [44–46]. This discrepancy may arise from participants from different cultural backgrounds having varying interpretations of the questionnaire items [46].

### The present study

The objective of this study was to develop a brief version of the AAQ tailored specifically for older Chinese adults (AAQ-BC) and examine its psychometric properties. The AAQ-BC aimed to fulfill several criteria: (1) it should be completed in a short amount of time, (2) it should capture attitudes toward aging from a multidimensional standpoint, and (3) it should be culturally appropriate for individuals with East-Asian backgrounds. We anticipated that this scale can serve as a standardized tool for measuring attitudes toward aging, convenient for use in nationwide social surveys.

## Methods

### Participants and procedure

#### Sample 1

The data were derived from the Chinese-version AAQ development study [33], in which 519 community-dwelling older adults were recruited from three cities in China (Beijing, Tianjin, and Chengdu). Among the 519 participants, 77 were excluded based on the following criteria: (1) age less than 60 years or missing age data (*n* = 28), and (2) at least one missing value in the AAQ items (*n* = 49). Thus, 442 participants were included (age: M = 68.62, SD = 5.55, range = 60–88).

#### Sample 2

The data were collected through a survey jointly launched by the Open University of China and Beijing Normal University in June 2021. This survey recruited a convenience sample of 713 Chinese adults who were enrolled in lifelong learning courses at the local college for senior citizens in three cities (Beijing, Ningbo, and Xinxiang). The participants completed a questionnaire during their spare time in the classroom. Participation in the survey was voluntary, and all participants provided verbal informed consent. For the 713 participants, the exclusion criteria were as follows: (1) age below 60 years or missing (*n* = 313), and (2) one or more missing responses on the AAQ items (*n* = 89). Thus, 311 participants were included in the analysis (age: M = 65.73, SD = 4.52, range = 60–90).

#### Sample 3

A total of 200 older adults were recruited from an urban community in Beijing by convenience sampling (April 2022). The participants were invited to participate in a face-to-face interview and were provided with a questionnaire to complete. Prior to their participation, all participants provided written informed consent. The exclusion criteria for the study sample were as follows: (1) age below 60 or missing (*n* = 5), and (2) one or more AAQ items missing (*n* = 31). Thus, 164 participants were included (age: M = 70.21, SD = 6.48, range = 60–89 years).

Out of the 164 participants, a test-retest sample of 29 individuals were selected at random (age: M = 68.03, SD = 4.72, range = 60–76). The participants completed the new AAQ format twice, with a time gap of two to three weeks between the tests. Owing to the temporary COVID-19 lockdown, both tests were administered via telephone.

The size of each sample met the recommended guideline of being at least 10 times larger than the number of variables used in the factor analysis [47]. Additionally, a power analysis indicated that the test-retest sample size of 29 participants was sufficient to achieve 0.80 power at α = 0.05, assuming Pearson *r* =.50. Individuals with severe cognitive impairments were excluded from the recruitment pool, based on a joint assessment by participants’ self-report and subjective evaluation by the investigators. Table 1 provides an overview of the characteristics of the study samples.

**Table 1:** Sample characteristics

Variable	Sample 1		Sample 2		Sample 3	
	(*n* = 442)		(*n* = 311)		(*n* = 164)	
	M/n	SD/%	M/n	SD/%	M/n	SD/%
Age						
60–69 years	250	56.56	249	80.06	89	54.27
70–79 years	176	39.82	58	18.65	56	34.15
80 years and above	16	3.62	4	1.29	19	11.59
Gender						
Men	225	50.90	78	25.08	49	29.88
Women	217	49.10	214	68.81	109	66.46
Not specified			19	6.11	6	3.66
Education						
Primary and below^a^	134	30.32	10	3.22	28	17.07
Middle school^b^	203	45.93	142	45.66	115	70.12
College^c^	102	23.08	154	49.52	21	12.80
Not specified^d^	3	0.68	5	1.61		
AAQ-BC						
PL (range: 4–20)	9.07	3.84	10.11	3.87	10.87	3.64
PC (range: 4–20)	12.68	4.14	15.34	3.27	15.73	3.53
PG (range: 4–20)	11.89	3.70	13.78	3.48	15.02	3.39

Note. AAQ-BC = the brief version of Attitudes to Ageing Questionnaire for older Chinese adults, PL = Psychosocial loss, PC = Physical change, PG = Psychological growth

^a^ Includes individuals with primary education or no formal education

^b^ Includes individuals with junior high school, vocational school, and high school education

^c^ Includes individuals with associate’s, bachelor’s, and graduate-level education

^d^ Includes participants who did not report their education level

### Measures

#### AAQ

The 24-item version of AAQ [31] was tested on Sample 1, and the new AAQ format (AAQ-BC) was tested on Samples 2 and 3. The full AAQ comprised three subscales (PL, PC, and PG), and each subscale contained eight items. A five-point Likert scale from 1 (strongly disagree) to 5 (strongly agree) was adopted for response. Scores were summed. Higher scores indicated more positive attitudes toward aging (PL was scored reversely). The item expression followed the Chinese-version AAQ [33]. Cronbach’s α ranged from 0.76 to 0.80 for subscales on Sample 1.

#### Measures for validity tests on sample 2

Depression was measured using a nine-item format of the Center for Epidemiologic Studies Depression Scale (CES-D) [48]. Participants were required to rate how often they experienced nine depressive symptoms over the past week using a three-point response format from 0 (hardly ever or never) to 2 (always). Scores were summed (range: 0–18), with higher scores indicating higher levels of depression (three items were scored reversely). This scale had demonstrated adequate reliability and validity among Chinese older people [49], and Cronbach’s α = 0.80 on Sample 2.

#### Measures for validity tests on sample 3

Depression was measured using a short-form CES-D [50, 51]. This scale consisted of 10 items, with responses coded from 0 (hardly ever or never) to 3 (always). Scores were summed (range: 0–30), with higher scores reflecting higher depression levels (two items were scored reversely). Internal consistency was adequate on Sample 3, with Cronbach’s α = 0.71.

Anxiety was measured using a brief version of the Generalized Anxiety Disorder Scale (GAD) [52]. This scale consisted of seven items. For each item, participants were asked to rate the frequency of experiencing the corresponding anxiety symptom in the last week. A four-point scale was adopted, with responses coded from 0 (not at all) to 3 (nearly every day). Scores were summed (range: 0–21), with higher scores indicating higher levels of anxiety. Internal consistency was adequate on Sample 3, with Cronbach’s α = 0.89.

Quality of life was evaluated via a single item taken from the World Health Organization Quality of Life-Bref assessment (WHOQoL-Bref): “How would you rate your quality of life?” [53]. Responses were coded from 1 (very poor) to 5 (very good).

Physical health was evaluated using two indicators: self-rated health (“How would you rate your physical health?”) and chronic disease (“Have you been diagnosed with any chronic disease?”). Participants rated their health status on a scale ranging from 1 (poor) to 5 (excellent) and responded to the question regarding chronic disease in a binary format (yes or no).

### Analytic plan

#### Overview

The analyses were conducted in a multiphase process. Initially, we selected items to create a new AAQ format using Sample 1. Once the format was determined, we carried out a parallel analysis to evaluate its reliability and validity using Samples 2 and 3.

Data analyses were performed using SPSS 27 unless otherwise specified. All tests were two-tailed, and the significance level was set at α = 0.05. Missing data were deleted listwise, so that the degrees of freedom could vary according to the different sample sizes.

#### Item selection

We planned to select 12 AAQ items to curate the new scale. This number of items would condense the scale length while averting excessive information loss. The procedure of item selection followed that used in the AAQ-SF development [40]. For each subscale, all items were ranked by item-total correlation coefficients (Pearson), and then the top four items with highest correlations were selected to form the new scale. This approach would ensure high consistency between the new scale and the full AAQ.

To evaluate the suitability of the selected items, we examined whether the new format preserved the original properties. First, we calculated the Pearson correlation coefficient between the new format and the original scale to evaluate scoring consistency. Correlation coefficients below 0.30 were considered small effect size, those ranging from 0.30 to 0.50 were considered medium effect size, and those above 0.50 were considered large effect size [54]. We expected a large-sized association between two scales. Second, we explored the factor structure of the new AAQ format. Kaiser-Meyer-Olkin index (KMO) and Bartlett test statistic were computed to evaluate the suitability of the data for factor analysis. Adequacy was indicated by a KMO value greater than 0.70 and a significant Bartlett test. The exploratory factor analysis (EFA) was performed using promax oblique rotation and principal component analysis. Instead of specifying the number of factors in advance, we retained factors with eigenvalues greater than one. Factor loadings greater than 0.30 would be considered significant. We expected all items to load onto their corresponding factors, while factor loadings less than 0.30 on all factors or greater than 0.30 on other factors would be considered as a poor fit.

#### Psychometric evaluation

First, we performed confirmatory factor analysis (CFA) on AMOS 24 to test construct validity according to the EFA results. The model fit was evaluated using a series of indices including chi-square to degree of freedom ratio (χ^2^/df), goodness-of-fit index (GFI), root mean square error of approximation (RMSEA), comparative fit index (CFI), and standardized root mean square residual (SRMR), where χ^2^/df < 3, GFI ≥ 0.90, RMSEA < 0.08, CFI ≥ 0.90, and SRMR < 0.10 indicated a good fit [55–57].

Next, we tested reliability including internal consistency and test-retest reliability. The Cronbach’s alpha coefficient was used to evaluate the internal consistency, with values higher than 0.70 indicating adequacy. Pearson correlation between the two telephone tests was used as the test-retest reliability estimate.

Then, we examined the convergent validity via computing Pearson correlations between the AAQ-BC scores between a series of external criterion variables including depression (two CES-D formats), anxiety (GAD-7), and quality of life (a single WHOQoL-Bref item). The AAQ-BC scores were expected to be significantly associated with these criterion scores.

Finally, we assessed the discriminant validity using independent t-tests. Effect size was measured using Cohen’s d, where values of 0.20, 0.50, and 0.80 indicated small, medium, and large effect sizes respectively [54]. It was expected that the AAQ subscales would perform differently in discriminating between groups with different health conditions.

## Results

### Item selection

Table 2 displays the results of item selection, showing the Pearson correlation coefficients ranging from 0.63 to 0.80.

**Table 2:** The results for item selection

Content	Pearson r
Psychosocial loss	
**12. I see old age mainly as a time of loss.**	**0.71**
**9. I find it more difficult to talk about my feelings as I get older.**	**0.69**
**15. I am losing my physical independence as I get older.**	**0.66**
**17. As I get older, I find it more difficult to make new friends.**	**0.63**
22. I feel excluded from things because of my age.	0.62
6. Old age is a depressing time of life.	0.60
20. I don’t feel involved in society now that I am older.	0.59
3. Old age is a time of loneliness.	0.56
Physical change	
**14. I have more energy now than I expected for my age.**	**0.80**
**23. My health is better than I expected for my age.**	**0.75**
**11. I don’t feel old.**	**0.69**
**16. Problems with my physical health do not hold me back from doing what I want to.**	**0.65**
8. Growing older has been easier than I thought.	0.63
24. I keep myself as fit and active as possible by exercising.	0.61
13. My identity is not defined by my age.	0.59
7. It is important to take exercise at any age.	0.40
Psychological growth	
**5. There are many pleasant things about growing older.**	**0.68**
**4. Wisdom comes with age.**	**0.66**
**2. It is a privilege to grow old.**	**0.66**
**18. It is very important to pass on the benefits of my experiences to younger people.**	**0.63**
1. As people get older they are better able to cope with life.	0.61
10. I am more accepting of myself as I have grown older.	0.60
21. I want to give a good example to younger people.	0.59
19. I believe my life has made a difference.	0.49

Note. *N* = 442. Twelve items selected for the AAQ-BC are in bold text

The scores obtained from the new AAQ format were strongly associated with those obtained from the full version, as indicated by a high correlation of 0.93 for the overall scale and 0.91–0.93 for the subscales (all *p* <.001).

The KMO = 0.81 and *p* <.001 for Bartlett’s test of sphericity indicated that the data were psychometrically fit for EFA. Three factors with eigenvalues greater than one were retained, accounting for 56.13% of the variation, which aligned with the three dimensions of the original scale. All items exhibited satisfactory factor loadings on their respective factors (Table 3).

**Table 3:** Results of the exploratory factor analysis

Item	Factor loading		
	1	2	3
Factor 1: Physical change			
14	**0.86**	0.01	0.02
23	**0.85**	0.07	− 0.08
11	**0.70**	− 0.12	0.05
16	**0.69**	− 0.02	− 0.01
Factor 2: Psychosocial loss			
15	− 0.05	**0.77**	− 0.02
9	0.04	**0.75**	0.03
12	0.08	**0.72**	− 0.04
17	− 0.10	**0.70**	0.04
Factor 3: Psychological growth			
4	− 0.09	− 0.04	**0.87**
2	− 0.09	0.01	**0.82**
5	0.28	− 0.01	**0.54**
18	0.22	0.10	**0.40**

Note. *N* = 442. The exploratory factor analysis was conducted using promax oblique rotation and principal component analysis. Factor loadings above 0.30 are in bold text

### Psychometric evaluation

**Fig. 1:**
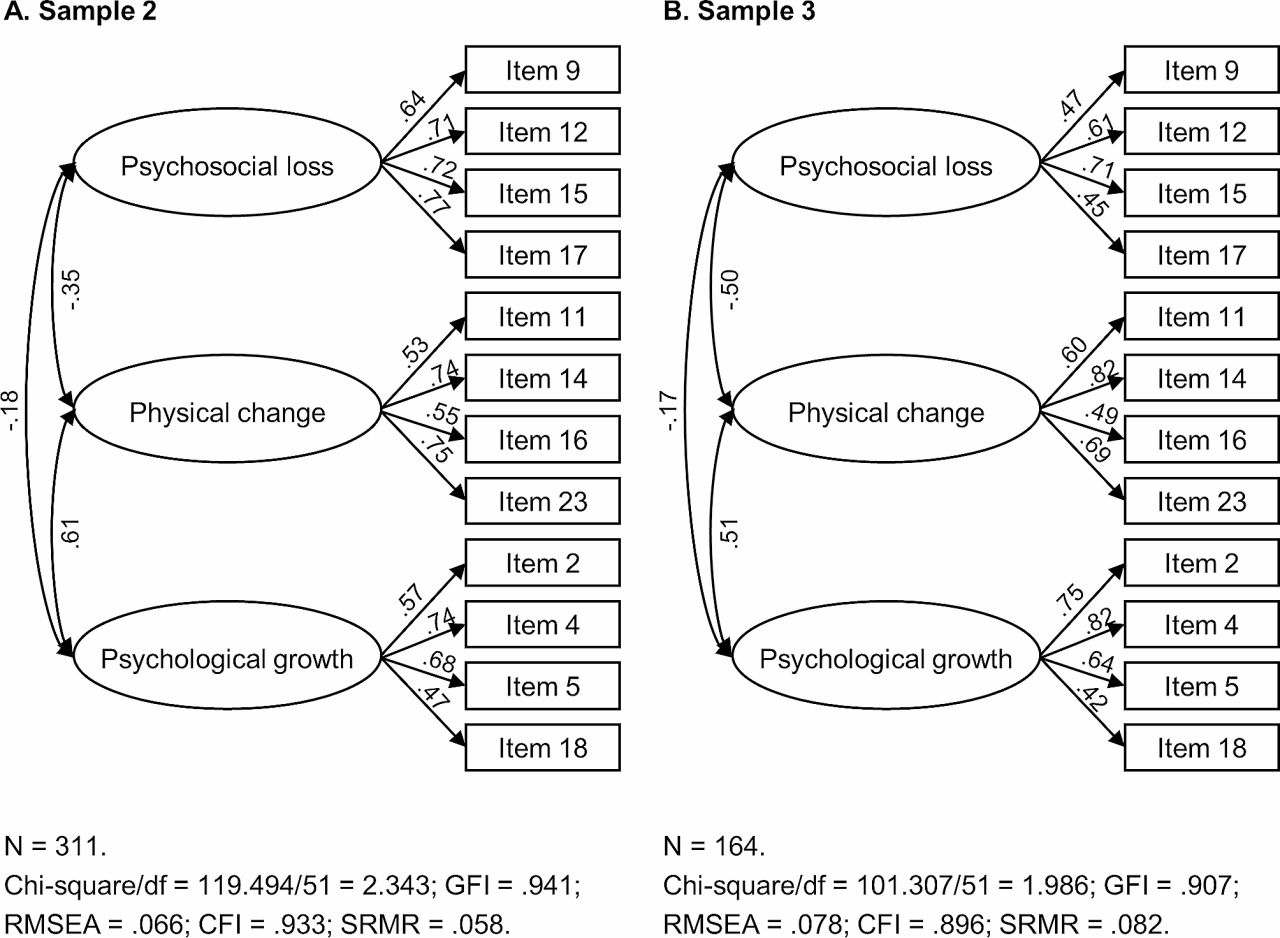
Results of Confirmatory Factor Analyses (Three-Factor Model) *Note.* Standardized coefficients are estimated. Residuals are not shown in the figure

Based on the results of EFA, we tested a model in which the 12 items of the AAQ-BC loaded onto their corresponding factors, allowing for correlations between the factors (Fig. 1). The model fit was found to be good for Sample 2, with χ^2^/df = 2.343, GFI = 0.941, RMSEA = 0.066, CFI = 0.933, and SRMR = 0.058. For Sample 3, the model fit was marginally acceptable, with χ^2^/df = 1.986, GFI = 0.907, RMSEA = 0.078, CFI = 0.896, and SRMR = 0.082. In addition to the three-factor model, we also considered two alternative solutions: (1) a single-factor model (1-factor), where all the 12 items loaded onto a single overarching factor; and (2) a hierarchical model (3 + 1 factor), where the 12 items loaded onto three factors, and these three factors loaded onto a second-order factor. Nonetheless, the single-factor model exhibited a poor fit. Moreover, the model fit of the hierarchical solution did not improve over the three-factor model, and the coefficients of the three factors predicted by the second-order factor were found to be imbalanced. Thus, we regarded the first-order three-factor model as the optimal solution. Refer to Table S1 and Figures S1 and S2 in the supplementary materials for additional details.

Reliability tests were performed for subscales. The internal consistency was adequate for Sample 2 (Cronbach’s α = 0.80 for PL, 0.73 for PC, and 0.71 for PG) and reached a marginally acceptable level for Sample 3 (Cronbach’s α = 0.65 for PL, 0.75 for PC, and 0.74 for PG). In addition, the test-retest reliability of all the subscales was found to be good, with Pearson correlation coefficients ranging from 0.77 to 0.88.

Table 4 presents the results of convergent validity tests. For both CES-D formats, more positive attitudes across all the subscales were significantly related to higher levels of depression for either sample (CES-D-9: Pearson *r* =.53, *p* <.001 for PL, Pearson *r* = −.38, *p* <.001 for PC, and Pearson *r* = −.27, *p* =.003 for PG; CES-D-10: Pearson *r* =.46, *p* <.001 for PL, Pearson *r* = −.46, *p* <.001 for PC, and Pearson *r* = −.39, *p* <.001 for PG). Similarly, for the GAD-7, more positive attitudes across all the subscales were significantly associated with higher levels of anxiety (PL: Pearson *r* =.38, *p* <.001; PC: Pearson *r* = −.30, *p* <.001; PG: Pearson *r* = −.23, *p* =.004). Moreover, more positive aging attitudes on the three subscales were linked to higher quality of life at least on a marginally significant level (PL: Pearson *r* = −.15, *p* =.060; PC: Pearson *r* =.42, *p* <.001; PG: Pearson *r* =.34, *p* <.001).

**Table 4:** Correlations between the subscale scores of AAQ-BC and criterion variables

Scale	N	M	SD	Pearson r		
				PL	PC	PG
Sample 2						
CES-D (9 items)^a^	302	4.04	3.41	0.53^***^	− 0.38^***^	− 0.27^***^
Sample 3						
CES-D (10 items)^b^	153	4.84	4.47	0.46^***^	− 0.46^***^	− 0.39^***^
GAD (7 items)^c^	159	2.91	3.44	0.38^***^	− 0.30^***^	− 0.23^**^
WHOQoL-Bref (1 item)^d^	164	4.18	0.81	− 0.15	0.42^***^	0.34^***^

Note. PL = Psychosocial loss, PC = Physical change, PG = Psychological growth, CES-D = Center for Epidemiologic Studies Depression Scale, GAD = Generalized Anxiety Disorder Scale, WHOQoL-Bref = the World Health Organization Quality of Life-Bref assessment

^a^ Uses a nine-item version of CES-D [49]

^b^ Uses a 10-item version of CES-D [50, 51]

^c^ Uses a seven-item version of GAD [52]

^d^ Uses a global item of WHOQoL-Bref: “How would you rate your quality of life?” [53]

^*^p <.05, ^**^p <.01, ^***^p <.001

Tables 5 and 6 present the results of discriminant validity tests. In terms of self-rated health, significant differences were observed between individuals who rated their health as “excellent” or “good” and those who rated their health as “fair”, “bad”, or “poor” in the PL and PC subscales (t = 3.28, *p* =.001, Cohen’s d = 0.53 for PL; and t = 4.85, *p* <.001, Cohen’s d = 0.78 for PC), while no significant difference was found in the PG domain (t = 1.22, *p* =.224, Cohen’s d = 0.19). Similar patterns of results were found when comparing participants with and without chronic disease (PL: t = 2.41, *p* =.017, Cohen’s d = 0.39; PC: t = 3.39, *p* <.001, Cohen’s d = 0.55; PG: t = 0.67, *p* =.504, Cohen’s d = 0.11).

**Table 5:** Results of t-tests differentiating the AAQ-BC scores among different self-rated health status groups

Subscale	Excellent/Good (*n* = 103)	Fair/Bad/Poor (*n* = 61)	t	Cohen’s d
PL	10.17 (3.64)	12.05 (3.34)	3.28^**^	0.53
PC	16.69 (3.12)	14.10 (3.60)	4.85^***^	0.78
PG	15.26 (3.59)	14.62 (3.02)	1.22	0.19

Note. Mean scores of the AAQ-BC subscales are reported. Standard deviations are in parentheses. PL = Psychosocial loss, PC = Physical change, PG = Psychological growth

^*^p <.05, ^**^p <.01, ^***^p <.001

**Table 6:** Results of t-tests differentiating the AAQ-BC scores between groups with and without chronic health conditions

Subscale	With chronic disease (*n* = 103)	Without chronic disease (*n* = 59)	t	Cohen’s d
PL	11.31 (3.53)	9.92 (3.58)	2.41^*^	0.39
PC	15.03 (3.63)	16.93 (3.06)	3.39^***^	0.55
PG	14.86 (3.48)	15.24 (3.29)	0.67	0.11

Note. Mean scores of the AAQ-BC subscales are reported. Standard deviations are in parentheses. PL = Psychosocial loss, PC = Physical change, PG = Psychological growth

^*^p <.05, ^**^p <.01, ^***^p <.001

## Discussion

The current study describes the development of a brief assessment tool (AAQ-BC) for measuring attitudes toward aging. This scale was tailored specifically for older Chinese adults, comprising 12 items taken from the full AAQ and demonstrating adequate reliability and validity.

The distinctions between the AAQ-BC format and the AAQ-SF [40] are intriguing. In our format, eight items were common with the AAQ-SF [40], while the other four items were different (items 4, 9, 15, and 16). We speculate that this might be due to the cultural difference between the two study samples. The inclusion of item 4 (wisdom comes with age) reflects the cultural values and beliefs surrounding aging in Chinese society. This item exhibited unclear factor loadings when administered in the Norwegian, French, and Portuguese populations [34, 36, 39]. In the Malay version, this item was removed, and the researchers regarded the act of respecting older adults in Malaysian society as a cultural norm rather than a reflection of their wisdom [38]. In Chinese historical anecdotes, older adults hold a revered position, often due to their embodiment of wisdom. Thus, wisdom is synonymous with psychological growth in old age. Additionally, the inclusion of item 9 (difficult to talk about my feelings) reveals the genuine challenges faced by Chinese older adults. In Chinese culture, it is not socially expected for older adults to express their feelings openly, as doing so can undermine their authority. In the process of combating ageism, encouraging them to speak out is of utmost importance. On the other hand, the exclusion of items related to disengagement (item 22) and exercise (item 24) may not fully capture the Chinese cultural perspective on active and healthy aging, where older Chinese adults prefer behind-the-scenes monitoring and “yangsheng” (means staying healthy with a balanced diet). Therefore, when compared to the AAQ-SF, our scale format appears to be more applicable within the cultural context of East Asia. Moreover, it is essential to recognize that our sample is relatively younger and exhibits more pessimistic attitudes on the PG subscale. These factors could also potentially influence the distribution of item scores.

Factor analyses identified a three-factor structure of the AAQ-BC, which remains consistent with the findings in previous studies [31–33, 36–40] and reaffirms its cross-cultural universality. Building upon this foundation, data results obtained from different versions of the AAQ can be cross-referenced. Furthermore, in line with the life-span development perspective [29], it is appropriate to assess the experiences of the aging process by considering both losses and gains, while recognizing that perceptions related to physical aspects tend to exhibit relative independence from psychosocial aspects. While some scholars have expressed doubts about the clarity of the three-factor structure or have argued that an overarching factor might be appropriate [34, 35, 37], our findings support the recommendation to report subscale scores rather than an overall score of the AAQ-BC. This aligns with the findings of Laidlaw et al. [40], suggesting that experiences in old age should not be measured using a single-dimensional scale. Hence, when evaluating an individual’s perception of aging as positive or negative, it is crucial to specify the particular aspect being assessed.

The scores of the AAQ-BC were found to be associated with external criterion variables, including depression, anxiety, and quality of life, and the PC subscale, as anticipated, exhibited the strongest correlation with physical health. These findings align with previous studies [34, 35, 37, 40], providing support for the satisfactory convergent and discriminant validity of the AAQ-BC. These findings underscore the pivotal role of positive attitudes toward aging in shaping late-life well-being. Notably, older adults have experienced stress during the COVID-19 pandemic, as their health faces greater threats [5], social media is inundated with offensive discourse (e.g., devaluing the lives of older people) [6], and some measures aimed at containing the virus may inadvertently result in new forms of ageism [7]. In such conditions, older adults are more likely to experience mental distress [8] and attribute the perceived deprivation to their own age. The development of the AAQ-BC serves as a valuable tool for addressing and healing this social crisis. It is noteworthy that negative experiences in the psychosocial domain might directly contribute to the mental distress of older individuals, as this dimension exhibits the strongest correlation with depression and anxiety. Therefore, the government should exercise caution when implementing social distancing policies, taking into account the risk of social decline that older adults face and providing remedies for the long-term impacts it may have on them.

### Theoretical and empirical implications

This study has several implications. Firstly, we provide a brief and multidimensional instrument for measuring attitudes toward aging of older Chinese adults. The AAQ-BC serves as a valuable tool for quick screening in large-scale social surveys, providing an overview of older individuals’ perspectives on their age. The dissemination of this tool will promote the utilization of the AAQ in a wider range of contexts, including assessing the effectiveness of interventions aimed at eliminating ageism. Secondly, this study contributes to providing explanations for beliefs about aging within the East-Asian cultural context and offering more effective and culturally sensitive support for older adults from this cultural background. Thirdly, the validity tests for the AAQ-BC further reinforce the significance of attitudes toward aging for the psychological well-being of older adults. Thus, this study underscores the importance of promoting and maintaining positive perceptions of aging among the older population and highlights the urgent need to combat ageism, which perpetuates negative age-related stereotypes. Finally, the findings suggest the adoption of a multidimensional approach when assessing the aging process, enabling a comprehensive understanding of older adults’ experiences and needs.

### Limitations and future development

Notably, there are several limitations that should be acknowledged in this study. Firstly, the data for analysis were obtained through convenience sampling. This approach was beneficial for collecting sufficient data during the COVID-19 pandemic, but it may limit the generalizability of the findings to the broader population. Thus, we conducted parallel analyses on two distinct samples of elderly individuals to ensure the robustness of the analytical results. Secondly, although the Cronbach’s alpha coefficient obtained for Sample 3 fell below the recommended threshold of 0.70, we consider the reliability of our scale acceptable based on similar alpha levels reported in previous studies [39, 40]. Thirdly, the test-retest data were collected during a period of COVID-19 lockdown, which placed older people under stress and led them to develop pessimistic perceptions of aging.

In future research, it would be beneficial to expand the sample pool to include individuals aged 80 and older, residents from rural areas, and other vulnerable groups. Additionally, we encourage the utilization of the AAQ-BC in other East-Asian countries. This would contribute to the promotion of our scale as a tool applicable to a broader range of older adult populations within the East-Asian cultural context. Furthermore, further exploration is needed to investigate the clinical application of the AAQ-BC and its potential utility in community interventions.

## Conclusions

The development and validation of the AAQ-BC were built upon previous research and followed classical psychometric methods. This process strikes a good balance between being concise and avoiding potential loss of information. The AAQ-BC is a reliable and valid assessment tool, which is applicable for assessing attitudes toward aging in Chinese older adult populations within social surveys that accommodate multiple questionnaires. This scale can provide assistance for policies aimed at eliminating ageism and enhancing older people’s well-being.

### Electronic supplementary material

Below is the link to the electronic supplementary material.

Supplementary Material 1

Supplementary Material 2

Supplementary Material 3

Supplementary Material 4

Supplementary Material 5

## Data Availability

The datasets supporting the conclusions of this article are included within the article (additional files: “Supplementary File 1.xlsx”, “Supplementary File 2.xlsx”, “Supplementary File 3_xlsx”, and “Supplementary File 4.xlsx”). Interested colleagues can obtain the data from the corresponding author (Dr. Dahua Wang, email: wangdahua@bnu.edu.cn) upon reasonable request. Moreover, additional details of confirmatory factor analysis can be found in “Supplementary File 5.docx”.

## Abbreviations

AAQ: Attitudes to Ageing Questionnaire
AAQ-BC: The brief version of Attitudes to Ageing Questionnaire for older Chinese adults
AAQ-SF: The shortened format of the Attitudes to Ageing Questionnaire
CES-D: The Center for Epidemiologic Studies Depression Scale
CFA: Confirmatory factor analysis
CFI: Comparative fit index
EFA: Exploratory factor analysis
GAD: The Generalized Anxiety Disorder Scale
GFI: Goodness-of-fit index
KMO: Kaiser-Meyer-Olkin index
PC: Physical change
PG: Psychological growth
PL: Psychosocial loss
RMSEA: Root mean square error of approximation
SRMR: Standardized root mean square residual
WHOQoL-Bref: The World Health Organization Quality of Life-Bref assessment
