# Supplementary material for: A brief version of the Attitudes to Ageing Questionnaire for older Chinese adults: development and psychometric evaluation
Source: BMC Psychol. 2024 Apr 1;12:181. doi: 10.1186/s40359-024-01691-z (PMC10986028; doi:10.1186/s40359-024-01691-z)
Supplement: Supplementary file 4 — Supplementary Material 4 [file 40359_2024_1691_MOESM4_ESM.docx]

# Supplementary Materials

In addition to the three-factor structure, we also considered two alternative solutions: (1) a single-factor model (1-factor), where all the 12 items loaded onto a single overarching factor (Figure S1); and (2) a hierarchical model (3+1 factor), where the 12 items loaded onto three factors, and these three factors loaded onto a second-order factor (Figure S2). Thus, we conducted CFA on both Samples 2 and 3. As depicted in Table S1, the model fit of the hierarchical solution did not improve over the three-factor model, while the single-factor model exhibited a poor fit.

**Table S1**

*The Evaluation for the Model Fit in Confirmatory Factor Analyses*

| Model | χ^2^ | df | χ^2^/df | GFI | RMSEA | CFI | SRMR |
| --- | --- | --- | --- | --- | --- | --- | --- |
| 3-factor (Sample 2) | 119.494 | 51 | 2.343 | .941 | .066 | .933 | .058 |
| 3-factor (Sample 3) | 101.307 | 51 | 1.986 | .907 | .078 | .896 | .082 |
| 1-factor (Sample 2) | 546.844 | 54 | 9.571 | .737 | .166 | .544 | .137 |
| 1-factor (Sample 3) | 237.054 | 54 | 4.390 | .780 | .144 | .621 | .115 |
| 3+1 factor (Sample 2) | 119.494 | 51 | 2.343 | .941 | .066 | .933 | .058 |
| 3+1 factor (Sample 3) | 101.307 | 51 | 1.986 | .907 | .078 | .896 | .082 |

*Note.* Sample 2: n = 311; Sample 3: n = 164. Indices for the model fit evaluation include chi-square to degree of freedom ratio (χ^2^/df), goodness-of-fit index (GFI), root mean square error of approximation (RMSEA), comparative fit index (CFI), and standardized root mean square residual (SRMR). 3-factor: the first-order three-factor model; 3+1 factor: the hierarchical model, where 12 items loaded onto three factors, and these three factors loaded onto a second-order factor; 1-factor: the single-factor model, where all 12 items loaded onto a single overarching factor.

**Figure S1**

*Results of Confirmatory Factor Analyses (the Single-Factor Model)*


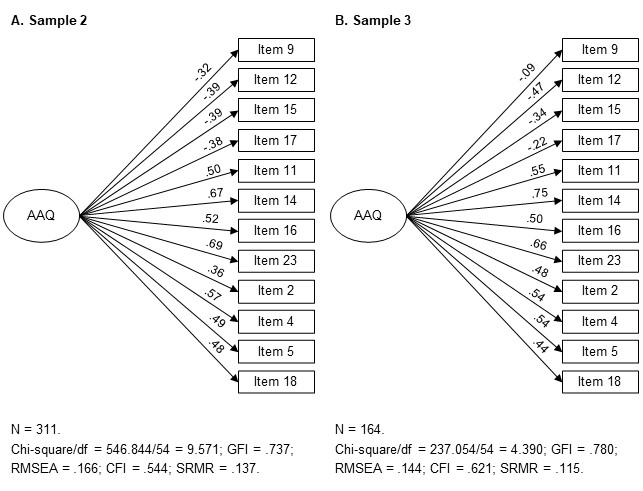


*Note.* Standardized coefficients are estimated. Residuals are not shown in the figure.

**Figure S2**

*Results of Confirmatory Factor Analyses (the Hierarchical Model)*


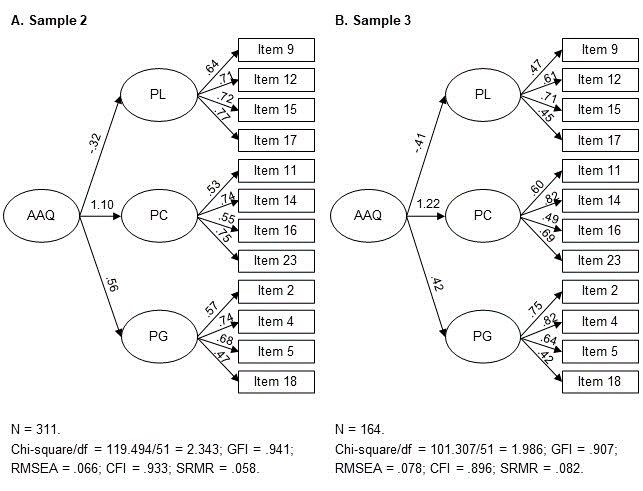


*Note.* Standardized coefficients are estimated. Residuals are not shown in the figure.
